# Trends and Characterization of Hospitalizations with Heart Failure in Italy Before and During the COVID-19 Pandemic

**DOI:** 10.3390/healthcare14111526

**Published:** 2026-05-30

**Authors:** Emanuele Amodio, Giovanni Tinervia, Sofia Bellomo, Michela Conti, Dario Genovese, Gabriele Biagio Marrella, Domenica Matranga, Aurelio Seidita, Giuseppe Vella, Marco Enea

**Affiliations:** 1 Department of Health Promotion, Mother and Child Care, Internal Medicine and Medical Specialties, University of Palermo, 90127 Palermo, Italy; emanuele.amodio@unipa.it (E.A.); giovanni.tinervia01@unipa.it (G.T.); sofia.bellomo@community.unipa.it (S.B.); dario.genovese@unipa.it (D.G.); gabrielebiagio.marrella@unipa.it (G.B.M.); domenica.matranga@unipa.it (D.M.); aurelio.seidita@unipa.it (A.S.); giuseppe.vella02@unipa.it (G.V.); 2 Unità Operativa Semplice Dipartimentale di Endocrinologia Adulti e ad Indirizzo Oncologico, Azienda Ospedaliera Ospedali Riuniti Villa Sofia-Cervello, 90146 Palermo, Italy; michelaconti@virgilio.it

**Keywords:** heart failure, hospitalizations, COVID-19, temporal trends analysis

## Abstract

**Highlights:**

**What are the main findings?**

- This 15-year national study analyzes over 6.2 million records, identifying advanced age, male sex, and winter seasonality as the strongest drivers of heart failure hospitalizations.
- While overall admission rates remained stable pre-pandemic, the data reveal a progressive increase in high-severity cases and a sharp, temporary drop in hospitalizations during 2020 due to healthcare disruptions.

**What are the implications of the main findings?**

- The growing burden of complex, severe cases and predictable seasonal peaks necessitates structured, multidisciplinary care models and flexible hospital capacity planning.
- Population-level data indicate that pandemic-era fluctuations were driven by organizational factors rather than COVID-19 infection or vaccination, underscoring the urgency of maintaining continuity of care during health emergencies.

**Abstract:**

**Background/Objectives:** Heart failure (HF) imposes a significant healthcare burden in aging populations. The COVID-19 pandemic disrupted care, raising concerns about chronic disease management. We analyzed temporal trends in HF hospitalizations in Italy (2008–2022), assessing the influence of demographics, clinical complexity, seasonality, and the pandemic. **Methods**: Using national discharge records, we strictly identified hospitalizations with a primary HF diagnosis via ICD-9-CM codes. Admissions were stratified by age, sex, season, and clinical severity according to the Elixhauser Comorbidity Index. Temporal trends were analyzed using a Negative Binomial Generalized Linear Mixed Model with the time component modeled through a segmented regression to account for pre-pandemic, pandemic (2020), and late-pandemic dynamics. **Results**: We identified 3,162,075 primary HF admissions, yielding a crude hospitalization rate of 35.11 per 10,000 person-years. Patients with an intermediate comorbidity burden (Elixhauser 13–20) accounted for 59.3% of the total volume. Multivariable analysis identified male sex (RR = 2.24, *p* < 0.001 ***), age ≥ 75 years (RR = 95.04 vs. 25–44, *p* < 0.001 ***), and winter seasonality as strong independent predictors. Trend analysis revealed a structural long-term decline across all severity tiers, driven by a sharp drop in 2020 (RR = 0.80, *p* < 0.001 ***) coincident with a spike in in-hospital mortality. While patients with low-to-intermediate comorbidity exhibited a partial rebound in 2021–2022 (overall RR = 1.06, *p* < 0.001 ***), admissions for highly complex patients (score > 20) showed an accelerated late-pandemic decline. **Conclusions**: HF hospitalizations in Italy remain a substantial burden driven by advanced age and clinical comorbidity. Our 15-year population-level data indicate no sustained, structural late-pandemic surge in HF admissions. The observed fluctuations were likely driven by severe healthcare disruptions and patient care avoidance rather than a true epidemiological shift, highlighting the urgent need for resilient chronic care systems during emergencies.

## 1. Introduction

Heart failure (HF) is a major public health challenge and a leading cause of hospitalization, disability, and healthcare resource utilization in high-income countries, particularly in aging populations such as Italy [1]. HF affects an estimated 64 million people worldwide [2], while in Europe, approximately 1–2% of the adult population lives with HF, with a median incidence of around 3.2 per 1000 person-years [3].

Despite advances in cardiovascular prevention and treatment, HF remains associated with high mortality, frequent exacerbations, and a substantial burden on the Italian National Health Service [4]. Clinically, HF is a heterogeneous syndrome resulting from structural and/or functional myocardial abnormalities that impair the heart’s ability to meet the metabolic demands of the body or do so only at the expense of elevated filling pressures. It represents the final common pathway of multiple cardiovascular conditions and reflects the interaction of cardiovascular, metabolic, and systemic factors [5]. HF prevalence increases markedly with age and is frequently accompanied by multiple comorbidities, contributing to clinical complexity and challenging long-term management [6].

HF has a profound impact on patients’ quality of life and is characterized by a chronic, progressive course with a high risk of recurrent hospitalizations [7,8]. Acute HF admissions are critical events in the natural history of this disease, often associated with worse prognosis, high risk of readmission and increased disability and social burden. From an epidemiological perspective, HF is a major contributor to global cardiovascular morbidity and mortality, accounting for a substantial proportion of years of life lost and years lived with disability [7,9].

Hospitalizations for HF represent a significant organizational and economic burden and are widely used as an indirect indicator of the quality and effectiveness of chronic disease management and continuity of care. High hospitalization rates may reflect not only the severity of the disease but also inadequacies in outpatient management and integrated care pathways. Recent international guidelines emphasize the necessity for structured, multidisciplinary follow-up aimed at preventing decompensation and avoidable hospital admissions [5].

The COVID-19 pandemic significantly disrupted healthcare systems, complicating access to care and service organization. Several studies have reported a marked reduction in hospital admissions for cardiovascular conditions during the acute phases of the pandemic [10,11], raising concerns about delayed care and suboptimal management of acute exacerbations of chronic diseases such as HF.

Based on this background, Italy represents a highly informative setting to investigate these dynamics, as it possesses one of the oldest demographic profiles worldwide, coupled with a universal National Health Service (SSN) that invests significantly in chronic disease management.

Therefore, the primary objective of this study is to analyze the long-term temporal trends of HF hospitalizations in Italy over a 15-year period (2008–2022), evaluating the impact of demographic factors, seasonality, and clinical complexity.

The secondary objective is to evaluate the structural impact of the COVID-19 pandemic on these trajectories. Specifically, beyond documenting the well-known acute drop in admissions during 2020, we hypothesized that the pandemic-induced healthcare disruption might have triggered a sustained, structural rebound in HF hospitalizations during the 2021–2022 recovery phase. By utilizing a national population-level dataset, we aim to explicitly test whether this major discontinuity event structurally altered the long-term epidemiological curve of heart failure.

## 2. Materials and Methods

This retrospective observational study examined hospitalization records in Italy from both public and private hospitals over a 15-year timeframe. Italy has an estimated resident population of approximately 60 million people, with one of the oldest demographic profiles worldwide [12], making it a relevant setting for the study of chronic, age-related conditions such as heart failure. Italy registers around 9 million hospitalizations annually [13], placing a significant burden on the healthcare system. This underscores the importance of monitoring hospital admissions for high-burden conditions such as HF. The Hospital Discharge Records database systematically collects demographic, clinical, and administrative information for all hospital admissions, including both inpatient and day-hospital, occurring within the Italian national territory.

The study included all hospitalizations occurring between 1 January 2008 and 31 December 2022, with HF recorded as primary diagnosis. Cases were identified using the International Classification of Diseases, Ninth Revision, Clinical Modification (ICD-9-CM) codes.

Specifically, admissions reporting the following diagnosis codes were considered eligible for inclusion:

- Heart failure: Codes 428.x (428.0–428.9).
- Rheumatic heart failure: Code 398.91.
- Hypertensive heart disease with heart failure: Codes 402.01, 402.11, 402.91.
- Hypertensive heart and chronic kidney disease with heart failure: Codes 404.01, 404.03, 404.11, 404.13, 404.91, 404.93.

The Diagnosis-Related Groups (DRG) approach was employed to stratify hospital admissions, enabling an assessment of clinical complexity and resource consumption for each hospitalization. The relative weight of each DRG served as a proxy measure for clinical case complexity and expected resource utilization [14]. To categorize this expected resource consumption, admissions were classified into four mutually exclusive categories based on the following DRG relative weight cut-offs: ≤1.05, 1.06–2.5, 2.5–5, and >5.

Furthermore, to robustly capture true clinical severity and systematically assess the burden of secondary conditions, the Elixhauser Comorbidity Index [15] was calculated for all hospital records. Based on this validated index, patient comorbidity profiles were stratified into three distinct tiers using the following score cut-offs: ≤12, 13–20, and >20.

Raw data were aggregated to calculate quarterly hospitalization rates. Additionally, to account for macroscopic shifts in overall healthcare system capacity, the total number of all-cause hospitalizations was calculated for each stratum. Stratification included the following variables: year, season (winter, spring, summer, autumn), sex, age group (≤14, 15–24, 25–44, 45–64, 65–74, ≥75 years), region of residence, DRG relative weight category, and clinical severity based on the Elixhauser Comorbidity Index. The average annual resident population for each demographic stratum, obtained from the Italian National Institute of Statistics (ISTAT), was used as the denominator for rate calculation [16].

Temporal trend analysis was performed using a Generalized Linear Mixed Model (GLMM), with a random intercept for the region of residence to account for geographical clustering and unobserved heterogeneity. Given the count nature of the data and the presence of overdispersion (assessed with an AIC-based comparison with an equivalent model but assuming a Poisson distribution), a Negative Binomial distribution with a logarithmic link function was assumed.

The model specification included fixed effects for season, sex, and age group to adjust for demographic confounding, alongside the natural logarithm of total all-cause hospitalizations to robustly account for macroscopic shifts in overall healthcare system capacity. To capture the temporal dynamics and the specific impact of the COVID-19 pandemic, we adopted a segmented regression framework. The time trend was modeled using a linear function with a structural break set in the year 2020. This formulation allowed for the estimation of three distinct components: (1) the baseline linear trend during the pre-pandemic period (2008–2019); (2) an immediate level change (step change) at the onset of 2020; and (3) a change in the slope (trend change) for the late-pandemic period (2020–2022). To evaluate differentiated temporal trajectories across patient subgroups, interaction terms were included between all time components (baseline slope, step change, and slope change) and both the DRG relative weight categories and the Elixhauser Comorbidity Index tiers. The model included the natural logarithm of the resident population as an offset term, converting absolute counts into population-based rates. Model estimates are reported in terms of hospitalization rate ratios (RR).

To quantify temporal trends in an interpretable manner, we calculated the Annual Percent Change (APC) for both the pre-pandemic period (2008–2019) and the pandemic/late-pandemic period (2020–2022), as well as the Average Annual Percent Change (AAPC) for the entire 15-year study period [17]. To prioritize the assessment of true clinical complexity over administrative factors, the APC and AAPC were computed specifically for the Elixhauser Comorbidity Index strata. To statistically achieve this, temporal estimates for the Elixhauser Comorbidity Index strata were averaged across the DRG classifications. This approach effectively isolates the temporal dynamics of true clinical complexity from administrative variations in expected resource consumption.

To confirm the robustness of our methodological framework, two sensitivity analyses were conducted, with full results provided in the Supplementary Materials. First, to validate the assumption of a single structural break in 2020, alternative segmented regression specifications incorporating one, two, and three breakpoints at various historical time points were systematically tested. Model fit was evaluated using the Bayesian Information Criterion (BIC) to select the most parsimonious model that accurately captured pandemic-related shifts without overfitting non-COVID-related temporal variations. Second, to account for regional differences in the magnitude and dynamics of the pandemic’s impact, the base Generalized Linear Mixed Model was expanded. Beyond the regional random intercept, we introduced region-specific random slopes for all temporal components (the pre-pandemic baseline slope, the 2020 step change and slope change). This advanced specification allowed for the estimation of differentiated, region-specific temporal trajectories, ensuring that the aggregated national estimates were robust against unobserved geographical heterogeneity.

All statistical analyses were conducted using R Statistical Software (version 4.5.0).

## 3. Results

Over the 15-year study period (2008–2022), a total of 3,162,075 hospitalizations with HF diagnosis were recorded in Italy, corresponding to an overall crude hospitalization rate of 35.11 per 10,000 person-years (p.y.) (Table 1). The demographic analysis revealed a slight male preponderance (rate: 36.31 vs. 33.97 per 10,000 p.y. for females). Age emerged as the strongest determinant of hospitalization risk, with rates being negligible in younger populations (fewer than 1.5 per 10,000 person-years for individuals under 45 years) but increasing exponentially with advancing age. In the 45–64 age group, the rate was 12.10 per 10,000 person-years, rising to 58.04 in the 65–74 age group, and ultimately peaking at 228.31 per 10,000 person-years in patients aged ≥ 75 years. A distinct seasonal pattern was observed throughout the study period. Hospitalization rates peaked during winter months (39.24 per 10,000 person-years), declined progressively through spring (38.22) and autumn (33.60), and reached their lowest point in summer (29.35). Regarding clinical complexity and expected resource utilization, admissions were stratified using both the Elixhauser Comorbidity Index and DRG relative weight categories. Based on the clinical comorbidity profile, the largest proportion of admissions (59.3% of total cases) fell into the Elixhauser score tier of 13–20 (rate: 20.80 per 10,000 person-years). Administratively, the majority of cases were classified within the DRG relative weight category of ≤1.05 (rate: 30.90 per 10,000 person-years), representing the core burden of HF patients requiring standard inpatient care.

**Figure 1:**
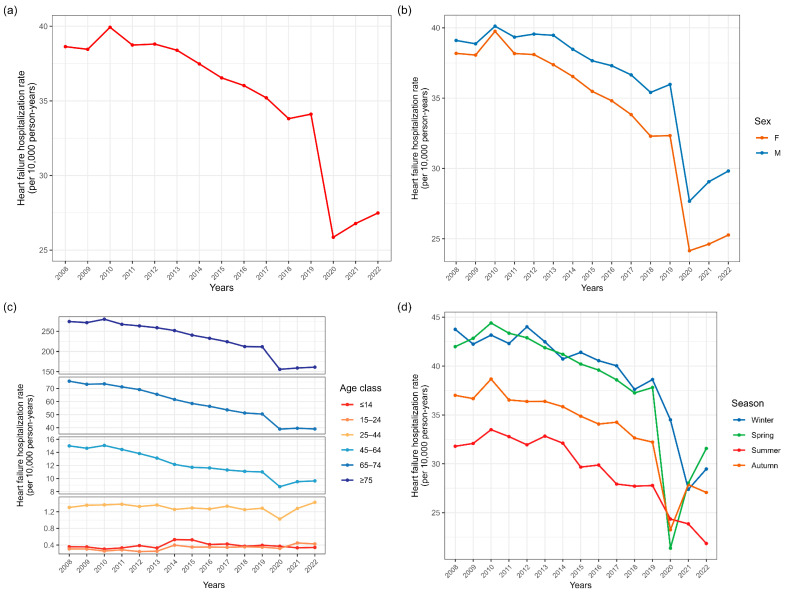
**Overall** observed temporal trends in heart failure hospitalization rates in Italy, 2008–2022 (**a**), and stratified by sex (**b**), age class (**c**), and season (**d**).

The overall unadjusted hospitalization rate (Figure 1a) showed a slight decline in the pre-pandemic period, fluctuating around 34 and 40 per 10,000 p.y. between 2008 and 2019. A sharp structural break occurred in 2020, with rates dropping to 26 per 10,000 p.y. (−24% compared to 2019), followed by a partial recovery in 2021 and 2022.

Stratified demographic analyses revealed distinct patterns across subgroups (Figure 1b–d). Trends for males and females proceeded in parallel throughout the study period (Figure 1b), with males consistently exhibiting higher hospitalization rates and maintaining a constant gap before experiencing a similar magnitude of decline during the 2020 pandemic onset. A consistent downward trend during the pre-pandemic period was observed across all adult age groups (Figure 1c), including both the intermediate classes (45–64 and 65–74 years) and the oldest cohort (≥75 years), which bears the majority of the disease burden. Conversely, hospitalization rates for younger cohorts (≤44 years) remained extremely low and substantially flat over the entire study period. The sharp reduction in 2020 visibly impacted all adult age classes, followed by a partial stabilization or slight rebound in 2021 and 2022. The seasonal pattern remained consistent throughout the study period (Figure 1d), with Winter consistently exhibiting the highest admission rates. The decline in 2020 impacted all seasons; however, the cyclical pattern was promptly restored in the subsequent years.

**Figure 2:**
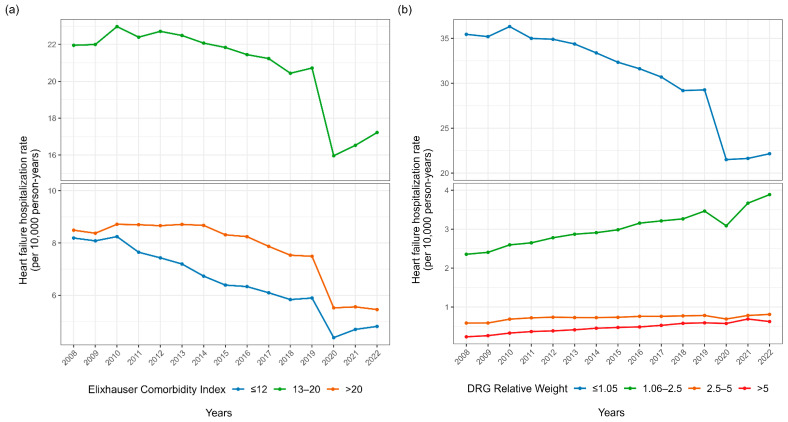
Observed temporal trends in heart failure hospitalization rates in Italy, 2008–2022, stratified by Elixhauser Comorbidity Index (**a**) and DRG Relative Weight (**b**).

Regarding clinical complexity, temporal trends were stratified by the Elixhauser Comorbidity Index and DRG Relative Weight (Figure 2). Analysis by the Elixhauser index (Figure 2a) revealed that patients with an intermediate comorbidity burden (score 13–20) consistently accounted for the highest hospitalization rates. All three comorbidity classes exhibited a general downward trend during the pre-pandemic period, followed by the sharp 2020 drop and a subsequent partial rebound. Stratification by DRG Relative Weight (Figure 2b) highlighted divergent trajectories: while hospitalizations for lower-complexity cases (DRG weight ≤ 1.05) progressively declined over the study period, admissions associated with higher expected resource consumption (DRG weight > 1.05) exhibited a steady, continuous upward trend, which was only temporarily interrupted by the 2020 pandemic onset.

**Figure 3:**
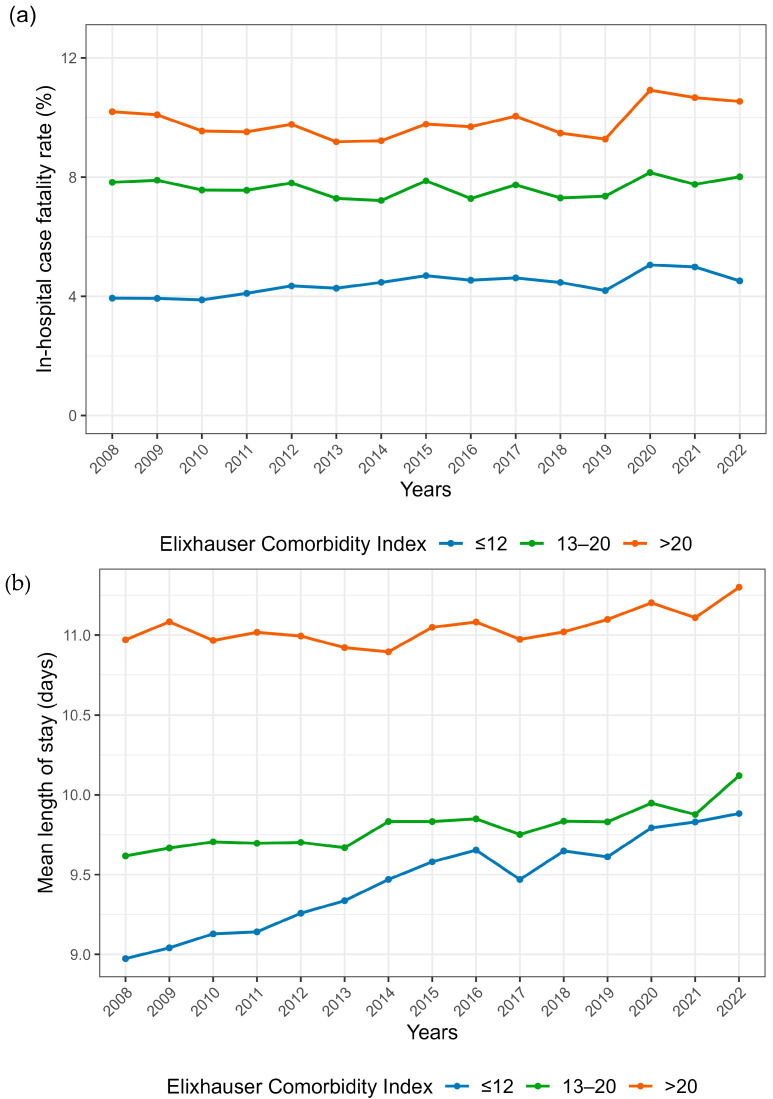
Observed temporal trends in in-hospital outcomes for heart failure in Italy (2008–2022), stratified by the Elixhauser Comorbidity Index: (**a**) In-hospital case fatality rate (%); (**b**) Mean length of stay (days).

To further contextualize the clinical burden and evaluate inpatient outcomes over time, we analyzed in-hospital case fatality rates and the mean length of stay (LOS), stratified by the Elixhauser Comorbidity Index (Figure 3). The in-hospital case fatality rate (Figure 3a) remained relatively stable during the pre-pandemic period, maintaining a clear and expected stratification based on patient complexity. Notably, in 2020, coincident with the pandemic onset, a visible spike in mortality occurred across all severity tiers, peaking at roughly 11% for the highly comorbid group (Index > 20), before showing a slight reduction in 2021–2022. Similarly, the mean LOS (Figure 3b) was strictly correlated with the comorbidity burden. Furthermore, LOS exhibited a steady, progressive upward trend over the entire 15-year study period across all patient subgroups, with the most complex patients consistently requiring the longest hospitalizations (averaging around 11 days).

Multivariable analysis (Table 2) confirmed that male sex (RR 2.240, 95% CI 2.215–2.26, *p* < 0.001) and advancing age (≥75 years: RR 95.036, 95% CI 91.988–98.185, *p* < 0.001) were strongly associated with higher risk of heart failure hospitalization. Notably, the rate ratio for the oldest cohort has a large value due to the extremely rare occurrence of heart failure in the young adult reference group (25–44 years). Seasonal effects were protective, with lower hospitalization risk observed in Summer (RR 0.819, 95% CI 0.808–0.830, *p* < 0.001) and Autumn (RR 0.913, 95% CI 0.901–0.924, *p* < 0.001) compared to Winter.

Regarding clinical complexity, hospitalizations were more than twice as frequent among patients with an intermediate Elixhauser Comorbidity Index (score 13–20: RR 2.695, 95% CI 2.633–2.758, *p* < 0.001) compared to the lowest comorbidity group (≤12). Conversely, higher DRG Relative Weights were inversely associated with hospitalization frequency (e.g., DRG 1.06–2.5: RR 0.090, 95% CI 0.088–0.092, *p* < 0.001 vs. ≤1.05), reflecting the smaller proportion of highly resource-intensive admissions within the overall volume.

The inclusion of the logarithm of total hospitalizations as a covariate (RR 1.179, 95% CI 1.148–1.210, *p* < 0.001) allowed the model to effectively adjust for macroscopic shifts in overall hospital activity and organizational capacity. Furthermore, the inclusion of a random intercept for the region of residence revealed a moderate degree of geographical heterogeneity in hospitalization rates (Standard Deviation = 0.47, 95% CI 0.34–0.68). This suggests that unmeasured regional factors, likely related to different healthcare organizational models and territorial care availability, influence admission patterns even after adjusting for demographic and clinical variables.

Temporal analysis revealed a declining trend in the pre-pandemic period (RR 0.949, 95% CI 0.945–0.952, *p* < 0.001), a sharp reduction in 2020 (RR 0.804, 95% CI 0.771–0.839, *p* < 0.001), and a late-pandemic rebound (RR 1.062, 95% CI 1.032–1.092, *p* < 0.001).

**Figure 4:**
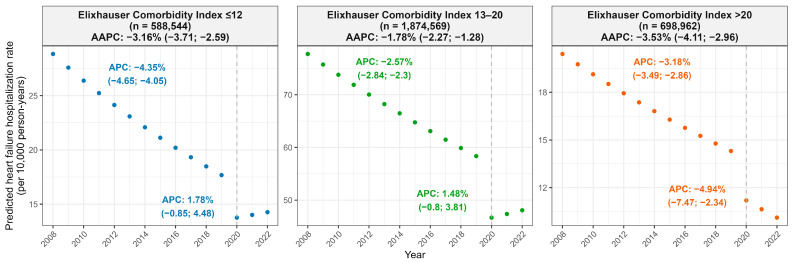
Predicted heart failure hospitalization rates (2008–2022) segmented by the 2020 pandemic onset. Plotted values represent estimated marginal trends for a reference profile (male, ≥75 years, Autumn), adjusted for DRG weights. The dashed grey line highlights the year 2020. Note: The selected covariates solely shift the *y*-axis scale; temporal slopes (APC/AAPC) remain constant across the population.

As reported in Figure 4, admissions across all Elixhauser Comorbidity Index categories exhibited a significant long-term decline over the 15-year study period. Hospitalizations for patients with the lowest comorbidity burden (Index ≤ 12) showed an overall Average Annual Percent Change (AAPC) of −3.16% (95% CI −3.71 to −2.59). This trajectory was driven by a steep reduction in the pre-pandemic period (APC: −4.35%, 95% CI −4.65 to −4.05), followed by a non-significant rebound during the 2020–2022 period (APC: 1.78%, 95% CI −0.85 to 4.48).

A similar pattern was observed for patients with an intermediate comorbidity burden (Index 13–20), which represented the largest volume of admissions. This group exhibited a significant overall decline (AAPC: −1.78%, 95% CI −2.27 to −1.28), characterized by a steady pre-pandemic decrease (APC: −2.57%, 95% CI −2.84 to −2.30) and a subsequent non-significant late-pandemic recovery (APC: 1.48%, 95% CI −0.80 to 3.81).

Conversely, highly complex cases (Index > 20) displayed a distinctly different late-pandemic behavior. While this group also experienced an overall structural decline (AAPC: −3.53%, 95% CI −4.11 to −2.96) and a significant pre-pandemic reduction (APC: −3.18%, 95% CI −3.49 to −2.86), they showed no signs of recovery after 2020. Instead, the downward trend for these highly comorbid patients significantly accelerated in the late-pandemic phase (APC: −4.94%, 95% CI −7.47 to −2.34).

## 4. Discussion

Over the 15-year study period, HF hospitalizations in Italy remained a substantial and largely stable burden on the healthcare system, with more than three million admissions recorded, corresponding to a crude rate of 35.11 per 10,000 person-years. From a public health perspective, the long-run stability of HF admissions—despite evolving therapeutic options—supports the interpretation of HF hospitalization as a “system indicator,” reflecting both population aging and the effectiveness of chronic disease management pathways across care settings.

As expected, advancing age emerged as the strongest determinant of hospitalization risk, with rates increasing exponentially in older adults, particularly those aged ≥ 75 years, consistent with prior evidence on the age-related epidemiology of HF [5,6]. These findings align with global evidence that HF prevalence and burden rise steeply in older populations due to accumulating comorbidities, cardiac remodelling, and age-related physiological decline [18].

Male sex was associated with higher hospitalization rates, consistent with previous large-scale epidemiological studies showing sex-specific variation in HF incidence and outcomes. Men exhibit a higher propensity for developing HF with reduced ejection fraction, frequently associated with ischemic heart disease. Conversely, women are more predisposed to HF with preserved ejection fraction and hypertension-related pathology. This reflects underlying differences in cardiovascular risk profiles and pathophysiology between sexes [19]. In a large U.S. cohort of over 4.7 million HF hospitalizations, males accounted for a slightly higher proportion of admissions and exhibited poorer in-hospital outcomes compared to females, who tended to be older and displayed different comorbidity patterns [20]. Pathophysiological mechanisms contributing to these disparities include sex differences in cardiac remodelling, myocardial fibrosis, metabolic responses to stress, and hormonal influences, which can affect HF progression and clinical presentation [19].

Our results further confirm a robust seasonal pattern in HF hospitalizations, marked by peak rates in winter and a decline during spring and autumn, culminating in minimal levels in summer. This pattern has been consistently observed in other settings and is likely multifactorial [21,22]. In winter, colder temperatures induce increased sympathetic activation, peripheral vasoconstriction, and elevated blood pressure, imposing additional hemodynamic stress on compromised myocardium. The winter season is linked to a higher circulation of respiratory pathogens such as *influenzavirus*, which can trigger systemic inflammation, fever, and increased metabolic demand, precipitating HF decompensation and hospital admission [23]. These findings have immediate service-planning implications: winter preparedness for HF should be embedded within broader seasonal surge policies (e.g., capacity planning, community monitoring of vulnerable patients, rapid diuretic adjustment pathways, and integration with respiratory infection control measures). In addition, evidence suggests that influenza vaccination in HF is associated with improved outcomes and reduced hospitalization risk, supporting vaccination campaigns as a pragmatic population-level intervention aligned with HF winter peaks [24].

Regarding clinical complexity and resource utilization, our dual stratification provides critical insights. Administratively, the predominance of standard hospitalizations (DRG relative weight ≤ 1.05)—which account for the vast majority of admissions—indicates that most HF inpatient care involves patients of intermediate complexity, forming the core of hospital workload. In contrast, the steady pre-pandemic increase in admissions with higher expected resource consumption (DRG relative weights > 1.05) probably reflects a growing share of resource-intensive cases, likely driven by population aging, higher comorbidity burden, and therapeutic advances that prolong survival while maintaining high-risk profiles as already observed elsewhere [25]. This shift toward greater case complexity should be further monitored in the future since it could have important implications for hospital organization, resource allocation, and the planning of specialized HF care. In fact, the observed shift implies not only increased bed demand but also rising care intensity (e.g., higher dependency, multidisciplinary input, and post-discharge support). Furthermore, the incorporation of the Elixhauser Comorbidity Index allowed us to robustly capture the true clinical burden underlying these administrative classifications. Clinically, the majority of HF inpatient care (59.3% of total cases) involves patients with an intermediate comorbidity profile (Elixhauser score 13–20), making them more than twice as frequent as low-comorbidity cases (Elixhauser ≤ 12; RR 2.695). Notably, temporal trends varied significantly based on this clinical profile. While patients with an intermediate comorbidity burden experienced a partial rebound after the 2020 drop, admissions for highly complex patients (Elixhauser > 20) showed no signs of recovery, with their downward trend significantly accelerating in the late-pandemic phase. This interplay between administrative resource intensity and the evolving clinical complexity of the patient population strengthens the rationale for investing in structured HF disease-management programs, transitional care, and community follow-up to prevent deterioration and reduce recurrent admissions [26,27,28].

Finally, temporal trends revealed a clear impact of the COVID-19 pandemic. A sharp reduction in HF hospitalizations occurred in 2020, with a partial recovery in subsequent years. Stratified analyses based on the Elixhauser Comorbidity Index indicated that patients with low to intermediate comorbidity burdens (score ≤ 20) were heavily affected by the 2020 drop and experienced a subsequent partial late-pandemic rebound. In contrast, admissions for highly complex cases (score > 20) showed no signs of recovery, with their downward trend significantly accelerating in the late-pandemic phase. Overall, this pattern suggests that the disruption primarily affected lower- and intermediate-complexity admissions, likely reflecting patient reluctance to seek care due to fear of nosocomial contagion, reorganization of hospital services and deferral of non-critical admissions, rather than a true epidemiological shift in underlying HF incidence in the general population, which cannot be evaluated from hospitalization records alone. Unquestionably, the COVID-19 pandemic underlined the need for robust, resilient health systems to sustain chronic disease management during crises [28]. Widespread disruptions to routine care were observed—an international study across 32 countries found roughly 30 million “missed” hospitalizations in 2020–2021 relative to pre-pandemic trends [29]. Cardiovascular admissions dropped sharply, with circa 1.68 million fewer circulatory-disease hospitalizations in 2020 than expected. Heart failure care was notably impacted: in Italy, HF admissions fell by approximately 43% during the spring 2020 lockdown compared to 2019 [30], and other nations reported similar declines (e.g., a 23% decrease in acute HF admissions in a multicenter Polish study [31]). Consequences included sicker patients on presentation and higher in-hospital mortality—in Poland, HF in-patient mortality rose from 5.2% to 6.5% during the pandemic and exceeded 30% among those with COVID-19 [31]. These findings raise concerns about continuity of chronic HF care: lockdowns and infection fears likely led to deferred care, interrupted follow-up, and less effective self-management [32]. Multidisciplinary voices have since called for system reforms to ensure continuity of care for HF and other chronic conditions during emergencies—for example, by expanding remote monitoring, proactive outreach to high-risk patients, and adaptive care pathways that can mitigate the impact of service disruptions [32,33].

Notably, although concerns were raised that SARS-CoV-2 infection or COVID-19 vaccination could increase cardiovascular risk [34,35], our 15-year population-level data do not show a sustained, structural surge in HF hospitalization rates following the onset of the pandemic. The sharp decline in 2020, followed by a partial rebound in 2021–2022, mirrors patterns seen in other acute and chronic conditions. These fluctuations are consistent with temporary disruptions in healthcare delivery, patient avoidance of hospital care due to fear of contagion and deferred routine management rather than a true shift in the underlying HF incidence. Furthermore, the late-pandemic rebound occurred across all age, sex, and severity subgroups, suggesting that organizational and behavioral factors—such as reduced access to outpatient follow-up, postponed elective care, and changes in health-seeking behavior during the pandemic—may have been the predominant drivers of hospitalization trends.

Importantly, our ecological, administrative study design precludes drawing definitive causal inferences regarding the direct biological effects of the virus or vaccines at the individual level. Similarly, the inherent lack of granular pharmacological data in the national registry prevents us from investigating the specific pro-arrhythmic effects of certain treatments widely used during the early pandemic phases. Nevertheless, the absence of a steeper, sustained growth curve in the late-pandemic phase is a reassuring public health finding. These findings suggest that any potential individual-level cardiovascular risks, whether infection-mediated, vaccine-related, or drug-induced, associated with the COVID-19 era were not reflected in a measurable, macroscopic increase in the overall structural burden of HF hospitalizations on the national healthcare system.

Several limitations should be acknowledged. First, the reliance on administrative hospital discharge data over a 15-year timeframe makes the analysis inherently susceptible to unmeasured confounders and gradual shifts in coding practices or reimbursement policies. For instance, the database itself inherently lacks data on socioeconomic status and lifestyle factors. Furthermore, ICD-9-CM codes lack granular clinical information such as ejection fraction, laboratory values, in-hospital pharmacological treatments (including specific COVID-19 therapies and their potential pro-arrhythmic complications), or medication adherence, preventing us from reliably stratifying patients into specific HF phenotypes. Altogether, while our regression model robustly adjusts for major demographic variables, seasonality, and comorbidity burden, the absence of these unmeasured individual-level factors introduces a risk of residual confounding. Second, because the national Hospital Discharge Records database is strictly anonymized to comply with privacy regulations, it lacks a unique patient tracking identifier. Consequently, multiple hospitalizations occurring for the same patient are counted as separate, independent events. This precludes the calculation of individual-level metrics such as 30-day readmission rates or the tracking of longitudinal patient trajectories. Third, the study did not include outpatient or primary care data, meaning HF exacerbations successfully managed in the community or emergency department are not captured. Furthermore, due to the structural availability of consolidated national SDO data at the time of extraction, our study period ends on 31 December 2022. Consequently, the analysis captures the early post-acute recovery phase but cannot evaluate the definitive long-term stabilization of admission trends following the official end of the WHO pandemic emergency in 2023. Additionally, the SDO database does not record individual-level data regarding SARS-CoV-2 infection history or COVID-19 vaccination status, strictly precluding any clinical analysis of their potential direct biological effects on HF hospitalizations. Finally, while our ecological design robustly captures macroscopic population-level trends, it cannot definitively disentangle the relative contributions of individual biological susceptibility from systemic healthcare disruptions or potential denominator instability during the COVID-19 pandemic.

## 5. Conclusions

Despite the acknowledged possible methodological limitations, our findings highlight the substantial and evolving burden of HF hospitalizations in Italy, driven by demographic aging, sex differences, seasonal variation, and shifting clinical comorbidity profiles. Importantly, the analysis suggests that the COVID-19 pandemic era did not result in a sustained increase in HF admissions at the population level; rather, the observed fluctuations might be predominantly attributed to organizational and behavioral factors, such as service disruption and care avoidance. These results underscore the need for public health strategies and integrated care models that ensure continuity of HF management, optimize hospital resources, and mitigate the impact of potential future healthcare disruptions.

## Figures and Tables

**Table 1:** Descriptive characteristics of heart failure hospitalizations in Italy, 2008–2022.

		Hospitalization Cases	Person-Years	Rates × 10,000 p.y.
Total		3,162,075	900,752,318	35.11
Season	Winter	883,641	225,188,080	39.24
	Spring	860,749	225,188,080	38.22
	Summer	660,976	225,188,080	29.35
	Autumn	756,709	225,188,080	33.6
Sex	F	1,573,132	463,150,979	33.97
	M	1,588,943	437,601,339	36.31
Age Class	≤14	4707	122,562,120	0.38
	15–24	2949	89,050,229	0.33
	25–44	30,908	237,121,101	1.3
	45–64	309,814	256,147,143	12.1
	65–74	565,445	97,419,241	58.04
	≥75	2,248,252	98,452,484	228.36
Hospitalisation year	2008	230,319	59,619,290	38.63
	2009	230,899	60,045,068	38.45
	2010	240,948	60,340,328	39.93
	2011	234,872	60,626,442	38.74
	2012	230,483	59,394,207	38.81
	2013	229,140	59,685,227	38.39
	2014	227,798	60,782,668	37.48
	2015	222,138	60,795,612	36.54
	2016	218,544	60,665,551	36.02
	2017	213,295	60,589,445	35.2
	2018	204,483	60,483,973	33.81
	2019	204,024	59,816,673	34.11
	2020	154,240	59,641,488	25.86
	2021	158,636	59,236,213	26.78
	2022	162,256	59,030,133	27.49
DRG Relative Weight	≤1.05	2,782,456	900,752,320	30.9
	1.06–2.5	271,888	900,752,320	3
	2.5–5	65,471	900,752,320	0.7
	>5	42,260	900,752,320	0.5
Elixhauser Comorbidity Index	≤12	588,544	900,752,320	6.5
	13–20	1,874,569	900,752,320	20.8
	>20	698,962	900,752,320	7.8

**Table 2:** Results of the multivariable Negative Binomial Generalized Linear Mixed Model analyzing demographic factors, clinical severity and complexity, and segmented temporal trends of heart failure hospitalization rates in Italy (2008–2022). Estimates are reported in terms of hospitalization rate ratios (RR).

	RR	95% CI	*p*-Value
Season (ref: Winter)			
Spring	1.012	0.999, 1.025	0.0652
Summer	0.819	0.808, 0.83	<0.001 ***
Autumn	0.913	0.901, 0.924	<0.001 ***
Sex (ref: F)			
M	2.24	2.215, 2.266	<0.001 ***
Age (ref: 25–44)			
≤14	0.251	0.24, 0.262	<0.001 ***
15–24	0.254	0.24, 0.269	<0.001 ***
45–64	9.623	9.376, 9.877	<0.001 ***
65–74	40.745	39.884, 41.623	<0.001 ***
≥75	95.036	91.988, 98.185	<0.001 ***
Log-total hospitalization			
	1.179	1.148, 1.21	<0.001 ***
DRG Relative Weight (ref: ≤1.05)			
1.06–2.5	0.09	0.088, 0.092	<0.001 ***
2.5–5	0.026	0.025, 0.027	<0.001 ***
>5	0.011	0.011, 0.012	<0.001 ***
Elixhauser Comorbidity Index (ref: ≤12)			
13–20	2.695	2.633, 2.758	<0.001 ***
>20	0.708	0.689, 0.727	<0.001 ***
Temporal trends			
Pre-pandemic Annual Trend (2008–2019)	0.949	0.945, 0.952	<0.001 ***
Immediate Level Change (2020)	0.804	0.771, 0.839	<0.001 ***
Change in Trend Slope (2020–2022)	1.062	1.032, 1.092	<0.001 ***
**Random effect**	**Parameter**	**Estimate**	**95% CI**
Region (intercept)	Standard deviation	0.47	0.34, 0.68

Significance: *** *p* < 0.001.

## Data Availability

The raw data supporting the conclusions of this article will be made available by the authors on request.

## Supplementary Materials

The following supporting information can be downloaded at: https://www.mdpi.com/article/10.3390/healthcare14111526/s1, Table S1: Results of the multivariable Negative Binomial Generalized Linear Mixed Model analyzing demographic factors, clinical severity, and segmented temporal trends of heart failure hospitalization rates in Italy (2008–2022); Table S2: Analysis of temporal trends in heart failure hospitalization rates by clinical severity (2008–2022); Table S3: Sensitivity Analysis of Breakpoint Specifications; Figure S1: Regional vs. national Average Annual Percent Changes (AAPCs) in heart failure hospitalizations.

## Abbreviations

The following abbreviations are used in this manuscript:

HF	Heart Failure
RR	Rate Ratios
APC	Annual percent change
AAPC	Average annual percent change
