# Supplementary material for: Trends and Characterization of Hospitalizations with Heart Failure in Italy Before and During the COVID-19 Pandemic
Source: Healthcare (Basel). 2026 May 30;14(11):1526. doi: 10.3390/healthcare14111526 (PMC13257109; doi:10.3390/healthcare14111526)
Supplement: Supplementary file 1 [file healthcare-14-01526-s001.zip › healthcare-4262453-supplementary.pdf]

Supplementary Materials

S1. Model specification

The linear predictor for the mean parameter of the Negative Binomial model used in the analysis is the following:

$$log\left(\frac{\mu_{ij}}{o_{ij}}\right) = \beta_0 + u_j + \beta_1 I(Sex_i = \text{M}) + \beta_2 I(Age_i = \text{<14}) + \beta_3 I(Age_i = \text{15-24}) + \beta_4 I(Age_i = \text{45-64}) +$$
$$\beta_5 I(Age_i = \text{65-74}) + \beta_6 I(Age_i = \text{≥75}) + \beta_7 I\left(Season_i = \text{Spring}\right) + \beta_8 I(Season_i = \text{Summer}) +$$
$$\beta_9 I(Season_i = \text{Autumn}) + \beta_{10}\log\left(Total_{Hosp_{ij}}\right)\alpha_1 year_i + \alpha_2(year_i - 2020)_+ + \alpha_3 I(year_i \geq 2020) +$$
$$\sum_{k\,\in\{13-20,>20\}} I(Elix_i = k)\left[\gamma_{0,k} + \delta\gamma_{1,k} year_i + \delta\gamma_{2,k}(year_i - 2020)_+ + \gamma_{3,k} I(year_i \geq 2020)\right] +$$
$$\sum_{m\,\in\{1.06-2.5,2.5-5,>5\}} I(Drg_i = m)\left[\delta_{0,m} + \delta_{1,m} year_i + \delta_{2,m}(year_i - 2020)_+ + \delta_{3,m} I(year_i \geq 2020)\right]$$

where:

- i and j are the record and region indices, respectively;
- o_{ij} is the offset for the ij-th record (resident population);
- u_j is the random intercept component;
- α_1 is the slope for years 2008-2019 when Severity condition is equal to Not Severe;
- $\alpha_1 + \alpha_2$ is the slope for years 2020-2022 when Severity condition is equal to Not Severe;
- α_3 is the step change for year 2020 when Severity condition is equal to Not Severe;
- $\gamma_{0,k}$ and $\delta_{0,m}$ are the changes in the general intercept for the different Elixhauser Comorbidity Index and DRG Relative Weight categories, respectively;
- $\gamma_{1,k}, \gamma_{2,k}, \gamma_{3,k}$ and $\delta_{1,m}, \delta_{2,m}, \delta_{3,m}$ represent the deviations from the reference groups for, respectively: the baseline slope (2008–2019), the change in slope after 2020, and the step change at 2020.

The baseline variable values are:

- $sex = F$
- $Age = 25 - 44$
- $Season = Winter$
- $Elixhauser\ Comorbidity\ Index = \leq 12$
- $DRG\ Relative\ Weight = \leq 1.05$

The estimated model coefficients are reported in full detail in Table S1.

Table S1. Results of the multivariable Negative Binomial Generalized Linear Mixed Model analyzing demographic factors, clinical severity, and segmented temporal trends of heart failure hospitalization rates in Italy (2008–2022). Estimates are reported in terms of hospitalization rate ratios (RR).

	**RR**	**95% CI**	**p-value**
Season (ref: Winter)			
Spring	1.012	0.999, 1.025	0.0652 .
Summer	0.819	0.808, 0.83	<0.001 ***
Autumn	0.913	0.901, 0.924	<0.001 ***
Sex (ref: F)			
M	2.24	2.215, 2.266	<0.001 ***
Age (ref: 25-44)			
≤14	0.251	0.24, 0.262	<0.001 ***
15-24	0.254	0.24, 0.269	<0.001 ***
45-64	9.623	9.376, 9.877	<0.001 ***
65-74	40.74	39.884, 41.623	<0.001 ***
≥75	95.03	91.988, 98.185	<0.001 ***
Log-total hospitalization (per unit increase)	1.179	1.148, 1.21	<0.001 ***
DRG Relative Weight (ref: ≤1.05)			
1.06-2.5	0.09	0.088, 0.092	<0.001 ***
2.5-5	0.026	0.025, 0.027	<0.001 ***
>5	0.011	0.011, 0.012	<0.001 ***
Elixhauser Comorbidity Index (ref: ≤12)			
13-20	2.695	2.633, 2.758	<0.001 ***
>20	0.708	0.689, 0.727	<0.001 ***
Temporal trends			
Pre-pandemic Annual Trend (2008–2019)	0.949	0.945, 0.952	<0.001 ***
Immediate Level Change (2020)	0.804	0.771, 0.839	<0.001 ***
Change in Trend Slope (2020–2022)	1.062	1.032, 1.092	<0.001 ***
Interactions: Time x DRG Relative Weight<br>Difference in Pre-pandemic Trend (2008-2019)			
1.06-2.5	1.07	1.067, 1.074	<0.001 ***
2.5-5	1.054	1.049, 1.059	<0.001 ***
>5	1.11	1.103, 1.117	<0.001 ***
Interactions: Time x DRG Relative Weight<br>Difference in Immediate Level Change (2020)			
1.06-2.5	1.109	1.062, 1.158	<0.001 ***
2.5-5	1.071	1.005, 1.142	0.0342 *
>5	1.141	1.06, 1.228	<0.001 ***
Interactions: Time x DRG Relative Weight<br>Difference in Slope change (2020-2022)			
1.06-2.5	1.034	1.006, 1.064	0.0178 *
2.5-5	1.028	0.986, 1.071	0.191
>5	0.924	0.881, 0.969	0.0011 **
Interactions: Time x Elixhauser Comorbidity Index<br>Difference in Pre-pandemic Trend (2008-2019)			
13-20	1.019	1.015, 1.022	<0.001 ***
>20	1.012	1.008, 1.016	<0.001 ***
Interactions: Time x Elixhauser Comorbidity Index<br>Difference in Immediate Level Change (2020)			
13-20	1.009	0.964, 1.056	0.6943
>20	0.993	0.941, 1.047	0.796

Interactions: Time x Elixhauser Comorbidity Index			
Difference in Slope change (2020-2022)			
13-20	0.979	0.95, 1.008	0.154
>20	0.923	0.891, 0.955	<0.001 ***
Random effect	Parameter	Estimate	95% CI
Region (intercept)	Standard deviation	0.47	0.34, 0.68

Significance: *** $p < 0.001$; ** $p < 0.01$; * $p < 0.05$

S2. Calculation of Temporal Trends

To quantify the temporal dynamics of hospitalization rates, we calculated the Annual Percent Change (APC) for each period and the Average Annual Percent Change (AAPC) for the entire study duration. These indicators were derived from the estimated model parameters reported in the model specification above.

Since the model includes interaction terms for both the Elixhauser Comorbidity Index and DRG Relative Weights with the time components, the overall temporal trends for each clinical comorbidity profile were calculated by marginalizing over the administrative DRG categories.

Calculation of APC

Let $\theta_{pre,k}$ and $\theta_{post,k}$ denote the linear slopes (on the logarithmic scale) for the pre-pandemic (2008–2019) and pandemic/post-pandemic (2020–2022) periods, respectively, for a given Elixhauser Comorbidity Index $k \in \{\leq 12, 13-20, > 20\}$.

Let p_m be the empirical proportion of hospitalizations belonging to the DRG Relative Weight category $m \in \{1.06-2.5, 2.5-5, > 5\}$, used as weights for the marginalization.

Based on the linear predictor equation, the slopes marginalized over DRG categories are defined as follows:

For the **Pre-pandemic period (2008-2018)**:

$$\theta_{pre,k} = \alpha_1 + \gamma_{1,k} + \sum_{m \in \{1.06-2.5, 2.5-5, >5\}} p_m \delta_{1,m}$$

For the **Post-pandemic period (2020-2022)**:

$$\theta_{post,k} = (\alpha_1 + \alpha_2) + (\gamma_{1,k} + \gamma_{2,k}) + \sum_{m \in \{1.06-2.5, 2.5-5, >5\}} p_m (\delta_{1,m} + \delta_{2,m})$$

For the reference categories the interaction terms are equal to 0.

The APC represents the estimated average percentage change in the rate per year within each time segment. It is calculated as:

$$APC_{period,k} = [\exp(\theta_{period,k}) - 1] \times 100$$

The 95% Confidence Intervals (CI) for the APCs were computed transforming the 95% CI for the estimated slope $\theta_{period,k}$. The variance of the estimated slope was obtained from the variance-covariance matrix of the model parameters with the usual formula for variance of linear combination of coefficients:

$$Var(\theta_{period,k}) = c^T \Sigma c$$

where Σ is the variance-covariance matrix of the fixed effects and c is a vector of weights defining the linear combination of the coefficients for the specific marginalized slope.

Calculation of AAPC

To summarize the trend over the entire 15-year study period, the AAPC was calculated as a geometric weighted average of the segment-specific trends. Let $w_{pre} = \frac{12}{15}$ and $w_{post} = \frac{3}{15}$ be the weights proportional to the length of the pre-pandemic and pandemic/post-pandemic periods, respectively.

The average slope $\bar{\theta}_k$ for the k-th Elixhauser category is defined as:

$$\bar{\theta}_k = w_{pre} \times \theta_{pre,k} + w_{post} \times \theta_{post,k}$$

The variance of the average slope was computed considering the covariance between the estimators:

$$Var(\bar{\theta}_k) = w^2{}_{pre} \times Var(\theta_{pre,k}) + w^2{}_{post} \times Var(\theta_{post,k}) + 2 \times w_{pre} \times w_{post} Cov(\theta_{pre,k}, \theta_{post,k})$$

The AAPC is then derived as:

$$AAPC_k = [\exp(\bar{\theta}_k) - 1] \times 100$$

Confidence intervals for the AAPC were computed transforming the 95% CI for the estimated average slope.

Estimates of APC and AAPC are reported in Table S2.

Table S2. Analysis of temporal trends in heart failure hospitalization rates by clinical severity (2008–2022). The table reports the Annual Percent Change (APC) for the pre-pandemic and post-pandemic periods, and the Average Annual Percent Change (AAPC) for the entire study duration, along with 95% confidence intervals.

	Estimate	95% CI	p-value
≤12			
APC Pre-pandemic period (2008-2019)	-4.35	-4.65, -4.05	<0.001 ***
APC Post-pandemic period (2020-2022)	1.78	-0.85, 4.48	0.186
AAPC (overall)	-3.16	-3.71, -2.59	<0.001 ***
13-20			
APC Pre-pandemic period (2008-2019)	-2.57	-2.84, -2.3	<0.001 ***
APC Post-pandemic period (2020-2022)	1.48	-0.8, 3.81	0.206
AAPC (overall)	-1.78	-2.27, -1.28	<0.001 ***
>20			
APC Pre-pandemic period (2008-2019)	-3.18	-3.49, -2.86	<0.001 ***
APC Post-pandemic period (2020-2022)	-4.94	-7.47, -2.34	<0.001 ***
AAPC (overall)	-3.53	-4.11, -2.96	<0.001 ***

Significance: *** p < 0.001; ** p < 0.01; * p < 0.05

S3. Sensitivity analysis

To ensure the robustness of our methodological choices, we conducted two comprehensive sensitivity analyses focusing on the temporal placement of the structural breakpoints and the potential unobserved geographical heterogeneity of the pandemic's impact.

Sensitivity analysis S1: Structural Breakpoint Specification

Our primary model specified a single structural break in the year 2020 to capture the immediate impact of the COVID-19 pandemic. To validate this assumption and rule out the necessity of additional historical breakpoints, we systematically tested alternative segmented regression specifications incorporating 1, 2, and 3 breakpoints at various time points across the study period.

Model fit and complexity were evaluated using the Akaike Information Criterion (AIC) and the Bayesian Information Criterion (BIC). Given the vast sample size of our dataset spanning 15 years, the BIC applies a larger penalty for model complexity compared to the AIC. While the AIC tended to favor slightly more complex models (e.g., adding a breakpoint in 2011), our primary epidemiological objective was to evaluate pre- versus post-pandemic temporal trajectories. Introducing additional, non-COVID-related historical breakpoints would unnecessarily overcomplicate the model and dilute the main findings. Therefore, the BIC was identified as the most appropriate metric for model selection. As detailed in Table S3, the most parsimonious model featuring a single breakpoint in 2020 yielded the lowest (best) BIC, confirming it as the optimal specification to answer our core research question.

Table S3. Sensitivity Analysis of Breakpoint Specifications (Top 6 Models by BIC)

Breakpoints tested	BIC	AIC
2020	534996,73	534463,55
2011, 2020	535014,23	534360,33
2010, 2020	535020,27	534366,38
2012, 2020	535021,34	534367,45
2013, 2020	535024,34	534370,45
2014, 2020	535038,08	534384,19

Sensitivity Analysis S2: Regional Heterogeneity and Random Slopes

The base Generalized Linear Mixed Model (GLMM) detailed in the main text assumes a region-specific random intercept but uniform temporal slopes across the national territory. To rigorously account for regional variations in the magnitude and dynamics of the pandemic's impact, we estimated a highly parameterized model incorporating region-specific random slopes.

In this expanded specification, the linear predictor was modified to allow the pre-pandemic baseline slope, the 2020 step change, and the post-pandemic slope change to vary by region. This advanced specification successfully estimated differentiated temporal trajectories for each region. Crucially, the inclusion of these random slopes did not meaningfully alter the aggregated national APC and AAPC estimates, with differences limited strictly to the second decimal place. This confirms that the national aggregated estimates generated by our primary model are robust against unobserved geographical heterogeneity.

The region-specific Average Annual Percent Changes (AAPCs) calculated from this complex model are visually summarized in the forest plot (Figure S1). The regional estimates demonstrate high consistency with the aggregated national estimate in terms of trend direction across all Elixhauser comorbidity profiles. While there is some expected variation in effect size across regions, the only exception in trend direction is Valle d'Aosta. However, as this is by far the smallest Italian region, its estimates are inherently subject to higher statistical volatility and wider confidence intervals.

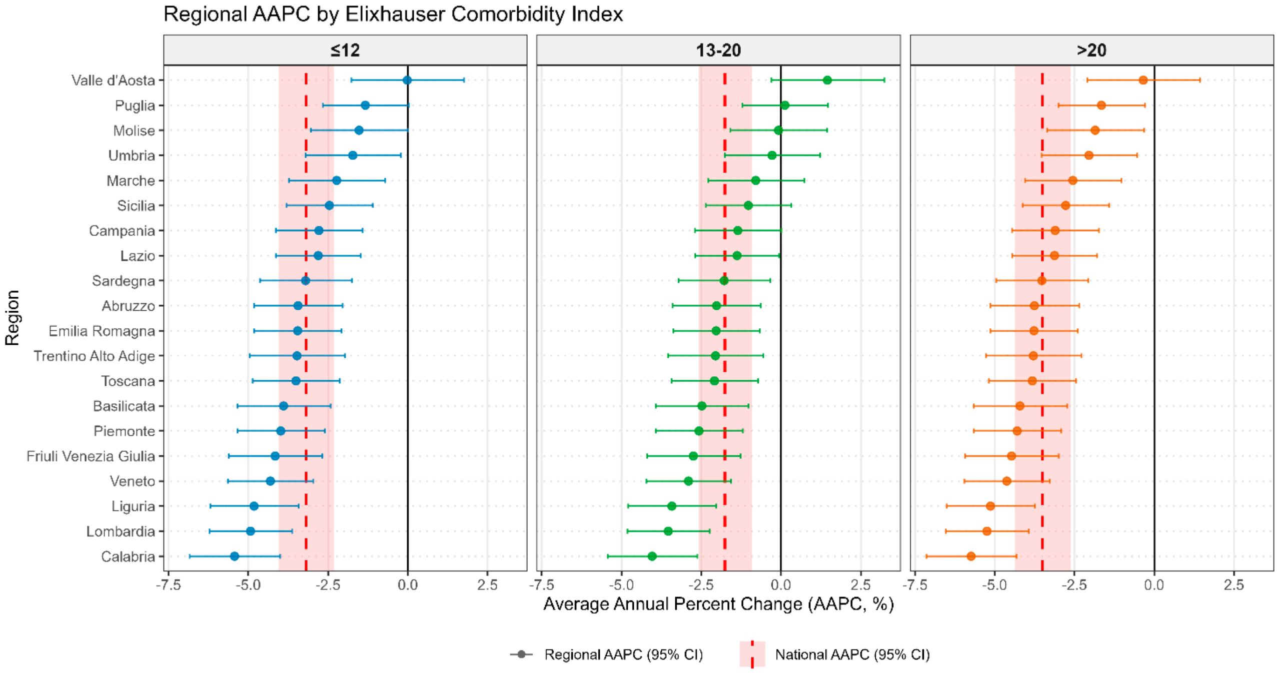

Figure S1. Regional vs. national Average Annual Percent Changes (AAPCs) in heart failure hospitalizations.
